# Monolithically multi-color lasing from an InGaN microdisk on a Si substrate

**DOI:** 10.1038/s41598-017-10712-4

**Published:** 2017-08-30

**Authors:** M. Athanasiou, R. M. Smith, J. Pugh, Y. Gong, M. J. Cryan, T. Wang

**Affiliations:** 1 Department of Electronic and Electrical Engineering, University of Sheffield, Sheffield, United Kingdom; 2 Department of Electrical and Electronic Engineering, University of Bristol, Bristol, United Kingdom

## Abstract

An optically pumped multi-color laser has been achieved using an InGaN/GaN based micro-disk with an undercut structure on a silicon substrate. The micro-disk laser has been fabricated by means of a combination of a cost-effective microsphere lithography technique and subsequent dry/wet etching processes. The microdisk laser is approximately 1 μm in diameter. The structure was designed in such a way that the vertical components of the whispering gallery (WG) modes formed can be effectively suppressed. Consequently, three clean lasing peaks at 442 nm, 493 nm and 522 nm have been achieved at room temperature by simply using a continuous-wave diode laser as an optical pumping source. Time–resolved micro photoluminescence (PL) measurements have been performed in order to further confirm the lasing by investigating the excitonic recombination dynamics of these lasing peaks. A three dimensional finite-difference-time-domain (FDTD) simulation has been used for the structure design.

## Introduction

Monolithically integrated multi-color lasers in the visible spectral region or white lasers are expected to find a wide range of applications, such as full colour high definition displays^1–6^, next generation vehicle headlighting, biosensors^7, 8^, visible light communications or even lighting^9^, etc. A number of approaches have been proposed in order to achieve white lasers. They are typically based on either a stacking structure or a structure containing multiple components, and are achieved exclusively by optical pumping, for examples, optical pumping a mixture of different fluorescence dyes in a gain medium^10^, optical pumping a mixture of II–V group semiconductor nano-particles with different diameters which can obtain multiple-emissions with different wavelengths in the visible spectral region^11^. More recently, an optically pumped white laser has been achieved on a stacking structure consisting of different II–VI group semiconductor layers^1^. These excellent results indicate that it is possible to achieve white lasing through a proper design. However, a new design is necessary in order to achieve electrically injected white lasers, which will be explained below.

The last two decades have seen major progress on developing III-nitride based visible laser diodes. The emission from an InGaN alloy can be tuned across the full visible spectral region by precisely controlling indium composition in InGaN quantum wells^12, 13^. III-nitrides exhibit major advantages compared with II–VI semiconductors in the visible spectral region. As a result, III-nitrides would be ideal candidates for achieving white lasers. Although III-nitrides demonstrate a huge potential for the fabrication of a white laser, a number of great challenges need to be overcome in order to realise a white laser. For example, it is difficult to fabricate a cavity which can support multiple-wavelength lasing simultaneously. The second challenge is due to carrier transport issues. For a stacked structure, injection current will have to go through different active regions where a threshold for lasing in each region needs to be met, leading to a challenge for achieving multiple colour lasing as a result of high injection current required (it might be sufficient for light emitting diodes where the injection current density required is not high). A simple solution which can address this issue effectively is to achieve very low threshold for lasing, thus easily meeting population inversion for different wavelengths. Unfortunately, it is well-known that III-nitride based lasers generally exhibit intrinsically high thresholds for population inversion or lasing.

In order to achieve III-nitride lasing with a low threshold, a number of approaches have been explored, such as photonic crystal laser^14^, plasmonic lasers, etc^15, 16^. However, these approaches typically require complicated fabrication processes in addition to a number of fundamental limitations. Fabrication into a micro-disk laser is an alternative option, and is actually becoming particularly important for III-nitrides^17, 18^. It leads to a significant reduction in threshold for lasing as a result of reduced modal volume. Furthermore, it does not require complicated processes for the fabrication of mirror facets, as optical confinement can be naturally achieved at the periphery of a micro-disk, forming well-known whispering gallery mode (WGMs) resonances. More importantly, a micro-disk laser can support a number of resonances, where both mode wavelengths and mode separation can be precisely tuned by controlling a micro-disk diameter. The mode separation of WGMs is given by equation 1 below

1 $${\rm{\Delta }}\lambda =\frac{{\lambda }^{2}}{2\pi Rn}$$

where λ is the wavelength, R the radius of the disk and n is the refractive index of the gain medium. Consequently, the radius of a micro-disk on a micrometre scale generates WGMs with a large mode spacing, which can span a wide range in the visible spectral regime that cannot be achieved in any conventional laser diodes. Note that a micro-disk also exhibits a large number of modes along a vertical direction as a result of optical confinement in the vertical direction. Therefore, a proper design is crucially important in order to effectively suppress the vertical modes.

These features make micro-disk cavities ideal for the fabrication of multi-color lasers due to their intrinsic abilities to simultaneously support resonances with a number of wavelengths, potentially forming white lasing with a low threshold. Therefore, these unique properties can meet the two major requirements mentioned above. Given the extremely mature and the cost effectiveness of silicon technology, a combination of III-nitrides with silicon technology makes them even more attractive for the realization of integrated photonics and electronics on the same chip. Recently, our group has demonstrated an optically pumped InGaN microdisk based green laser with a record low threshold operating in a continuous wave (CW) mode at room temperature^19^. The micro-disk laser was fabricated on an InGaN/GaN multiple quantum well (MQW) structure grown on a silicon substrate. The utilization of a silicon substrate can simplify the fabrication process as a result of ease in introducing an air gap by a chemical wet-etching technique.

In this study, we demonstrate a monolithic multi-color laser optically pumped at room temperature with a low threshold from a single InGaN microdisk on a Si substrate, which has never been achieved in the field of III-nitrides. The micro-disk laser has been fabricated by means of a cost effective microsphere lithography technique along with a proper design, demonstrating lasing at three distinct peaks, covering the blue, cyan and green spectral regions, namely, at 443 nm, 492 nm and 522 nm respectively. It is also worth highlighting that our structure design has effectively suppressed the vertical modes.

The micro-disk lasers used in the present study have been fabricated on an InGaN/GaN multiple quantum well epiwafer grown on (111) silicon by means of using a standard metal organic chemical vapor deposition (MOCVD) technique. An initial AlN buffer layer with a thickness of 400 nm was grown, followed by a 250 nm GaN buffer layer and then two different kinds of InGaN/GaN MQWs each with different indium content: first 3 pairs of InGaN/GaN MQWs with indium composition of approximately 18% with its emission at 450 nm; and then 5 pairs of InGaN/GaN MQWs with an emission at 530 nm, where the indium composition is approximately 28%. The thickness of the AlN buffer layer was designed and engineered in a way which helps to suppress higher order modes in the vertical direction, thus enhancing the confinement of the WGMs formed. This will be explained later through a simulation based on a three dimensional (3D) finite-difference- time-domain (FDTD) approach.

**Figure 1:**
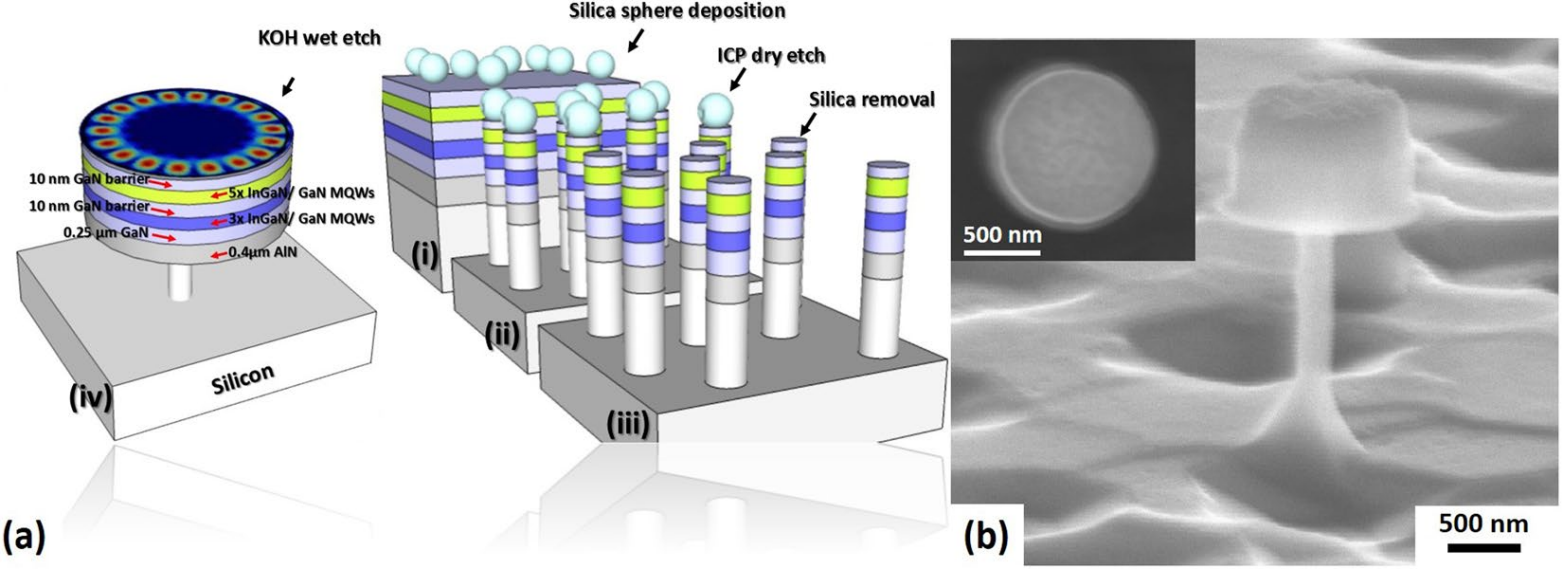
(**a**) Schematic illustration of our multi-color micro-disk laser, where Inset shows our procedure of the fabrication of dispersed micro-disks on a silicon substrate; **(b)** Side-view SEM image of our typical micro-disk with a diameter of ~1 μm, demonstrating straight and smooth sidewalls with a large air gap underneath.

Figure 1a schematically depicts our microdisk laser which is fabricated from the InGaN/GaN MQWs epiwafer. Figure 1a also shows a schematic illustration of our fabrication procedure using a microsphere lithography approach^20, 21^. Figure 1b shows a cross-sectional scanning electron microscopy (SEM) image of our microdisk, demonstrating straight sidewalls with a thin silicon post underneath which is left to mechanically support the microdisk. The microdisk is approximately 750 nm thick. An air gap formed underneath was introduced by a chemical wet-etching approach using a potassium hydroxide (KOH) solution, and the air gap is approximately 1.2 μm in height. It is expected that the introduction of the air gap underneath will enhance the optical confinement in the microdisk region. A top-view SEM image provided in the inset of Fig. 1b demonstrates an excellent circular geometry of our microdisk whose diameter is approximately 1 μm. It is worth highlighting that any imperfections or roughness at the periphery of the micro-disk will generate disruption to optical confinement as a result of scattering, leading to a leakage of photons out of the cavity and thus reducing its quality factor. This will result in an increase in threshold for lasing.

**Figure 2:**
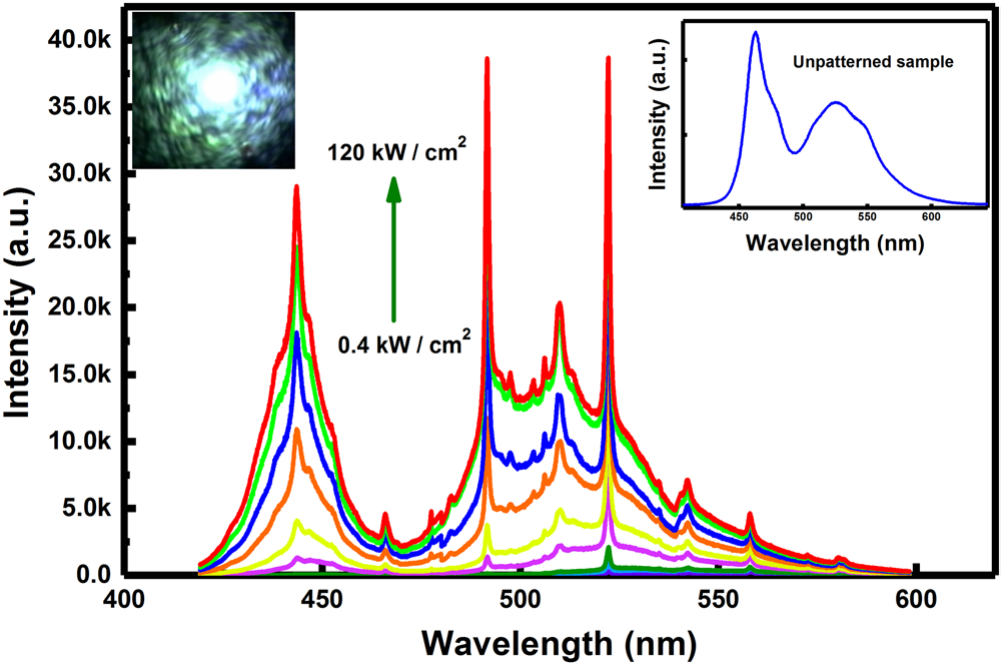
Lasing spectra from our micro-disk laser recorded as a function of optical pumping power density at room temperature, where Inset shows the emission spectrum of the un-patterned (i.e., as-grown) sample for comparison. Optical image of the micro-disk laser captured during optical excitation, demonstrating a white emission of the micro-disk laser.

Lasing spectral measurements have been performed on our microdisk laser at room temperature using a commercial confocal system, where a CW 375 nm diode laser is used as an optical pumping source and the spatial resolution of the system is around 160 nm. The system is equipped with a high resolution x-y-z piezo-stage, allowing us to preferentially excite and then collect any emission from a single micro-disk (see supplementary material). Figure 2 shows the emission spectra of our microdisk laser as a function of optical pumping density ranging from 0.4 to 120 kW/cm^2^. Under low optical pumping, a few weak emission peaks corresponding to different WGMs have been observed. However, when the optical pumping density is above the lasing threshold, three very sharp and strong emissions located at 443 nm, 492 nm and 522 nm have been observed. In each case, the intensity increases dramatically along with a significant reduction in full width at half maximum (FWHM) with further increasing optical pumping density, indicating a lasing action. The inset of Fig. 2 provides the emission spectra of the as-grown sample for comparison, showing two broad emission peaks from the two kinds of InGaN/GaN MQWs with different indium content as expected. Furthermore, Fig. 2 also shows a typical emission image captured when the optical pumping density is above the threshold, demonstrating multi-color lasing achieved on our micro-disk laser.

**Figure 3:**
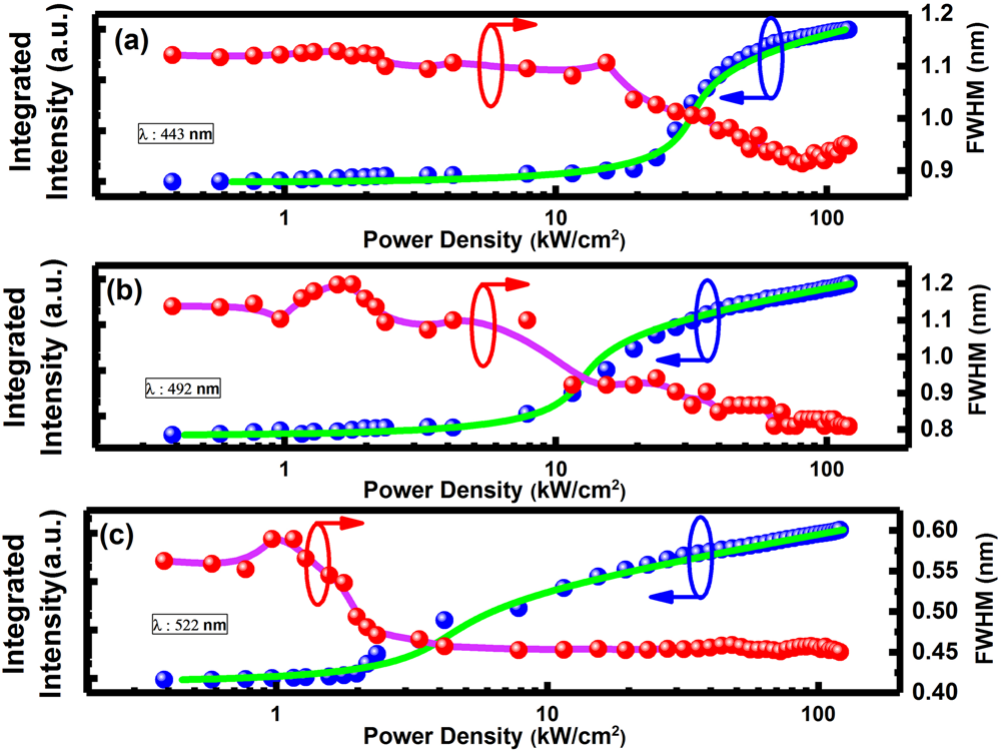
(**a**) to (**c**) Integrated emission intensity and FWHM plotted as a function of optical excitation power density for the three lasing peaks at 443 nm, 492 nm and 522 nm, respectively. A typical “s” shaped behavior of the integrated emission intensity has been observed along with a sudden reduction in FWHM in each case, confirming the lasing action.

Figure 3(a) to (c) show the light-light (L-L) curves of the three lasing peaks at 443 nm, 492 nm and 522 nm. In each case, the L-L curve plotted in a log-log scale exhibits a “s” shaped behaviour which is a typical fingerprint of lasing action demonstrating the three steps process towards lasing, namely, spontaneous emission, then amplified spontaneous emission, and final lasing oscillation. From the L-L plots, the thresholds are determined to be 27 kW/cm^2^, 15 kW/cm^2^ and 5 kW/cm^2^ for the lasing peaks at 443 nm, 492 nm and 522 nm, respectively. In fact, the actual thresholds are even lower, in particular for the blue peak, as only a small portion of optical pumping power can reach the blue MQWs region which is located below the green MQWs region as the optical pumping power needs to firstly pass through the green MQWs region. Figure 3(a) to (c) also provide the FWHMs of the three emission peaks as function of optical pumping density. In each case, the FWHM shows a dramatic reduction when the optical pumping density exceeds the threshold for lasing, further confirming the lasing behavior.

The quality factor (i.e., Q factor) which is defined as λ /Δλ can be determined, where λ and Δλ are the wavelength and its FWHM respectively. Under low optical pumping, the Q factors are ~ 500, ~ 620 and ~ 1200 for the three peaks at 443 nm, 492 nm and 522 nm respectively.

In order to further investigate the lasing properties of our micro-disk laser, time-resolved micro-PL (μ-TRPL) studies have been conducted as a function of excitation power density from 0.75 to 37 kW/cm^2^, which spans a wide range from a spontaneous region through a threshold to a lasing region. A 375 nm pulsed diode laser with a pulse width of 50 ps was used as an optical excitation source.

**Figure 4:**
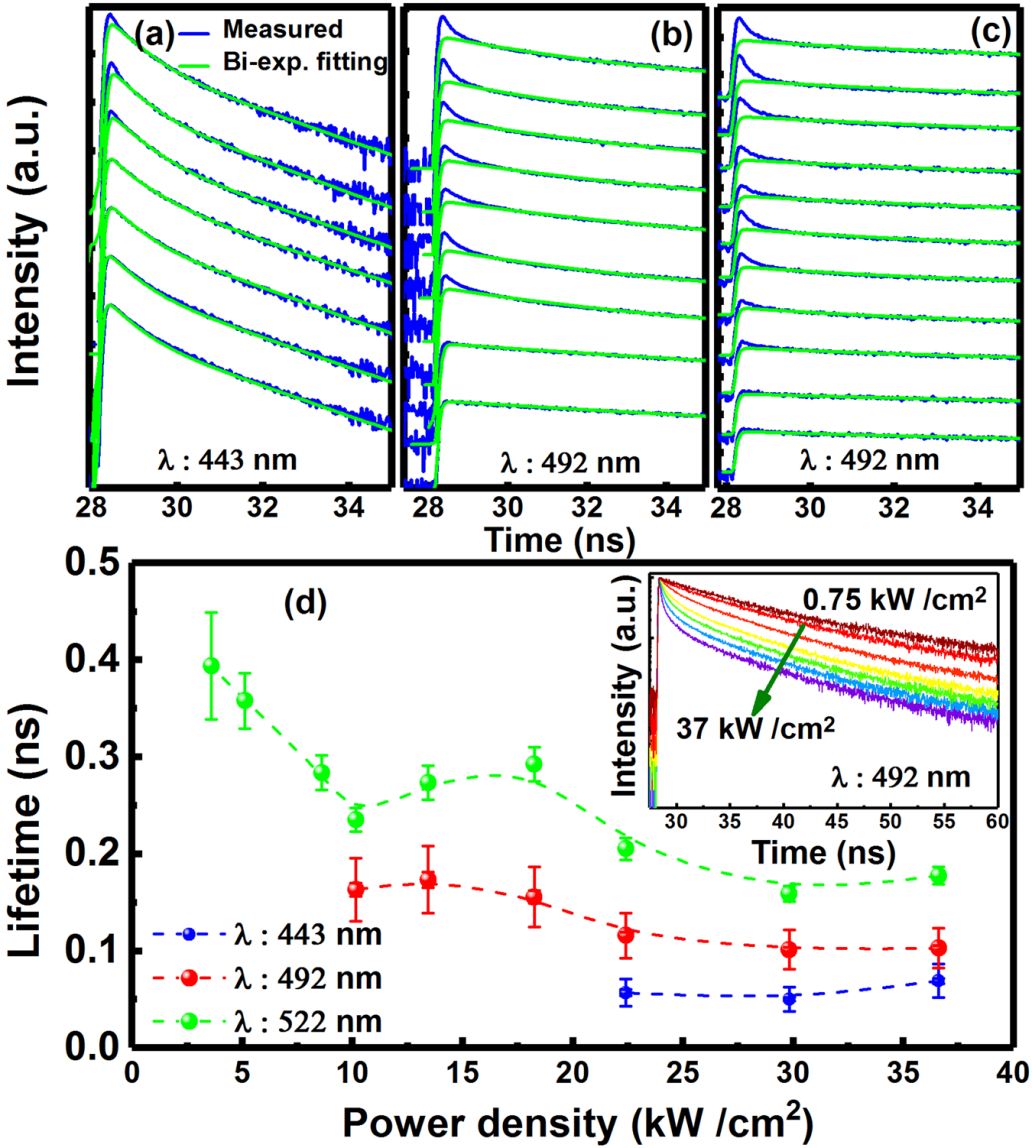
(**a**) to (**c**) μ-TRPL decay traces measured as a function of excitation power density for the three lasing peaks, where a standard bi-exponential mode, labelled as a green line, was deliberately used to fit the TRPL traces in order to highlight the evolution of the ultra-fast decay component under the lasing conditions; **(d)** Decay lifetime of the ultra-fast component in each case as a function of optical excitation power density, and Inset showing an example of the decay traces as a function of excitation power density.

Figure 4(a) to (c) shows the μ-TRPL decay traces measured as a function of excitation power density at room temperature. A standard bi-exponential model was used to fit the TRPL decay traces when the excitation power density used is below the threshold, and a TRPL trace labelled as I(t) can be described by Equation 2 provided below^19–21^:

2 $$I(t)={A}_{1}\exp (-\frac{t}{{\tau }_{1}})+{A}_{2}\exp (-\frac{t}{{\tau }_{2}})$$

where A_1_ and τ_1_ (A_2_ and τ_2_) represent the fast (slow) decay components^22, 23^. Figure 4(a) to (c) also provide the fitting data plotted as green curves, showing that a well-fitting has been obtained using the standard bi-exponential model for the TRPL-decay curves measured when the excitation power density is below the threshold.

However, when the excitation power is above the threshold, the situation has changed, and an extra ultra-fast decay component has been observed in each case. This can be observed more clearly after the data-fitting using the bi-exponential model (green lines). In this case, it is clear that the bi-exponential model no longer works for the TRPL traces, while an extra ultra-fast decay component is required in order to fit the TRPL traces. Therefore, an extra item needs to be added into Equation 2 as shown below:

3 $$I(t)={A}_{1}\exp (-\frac{t}{{\tau }_{1}})+{A}_{2}\exp (-\frac{t}{{\tau }_{2}})+{A}_{3}\exp (-\frac{t}{{\tau }_{3}})$$

where A_1_ and τ_1_ (A_2_ and τ_2_) remain unchanged as stated above, and the last term represents the extra component, which is due to lasing^19, 24^.

Figure 4d shows the decay lifetimes of the lasing (i.e., ultrafast component labelled as τ_3_) of the three peaks plotted as a function of excitation power density. As an example, the inset of Fig. 4d shows the TRPL decay curves of the emission peak at 492 nm recorded as a function of excitation power density, showing that the extra component starts to appear when the excitation power density is ≥10 kW/cm^2^.

Figure 4d indicates that the lifetimes of the ultra-fast decay components for the three lasing peaks are all on the order of 10 s ps. In detail, they are around 70 ps, 100 ps and 180 ps for the lasing peaks at 443 nm, 492 nm and 522 nm under the highest excitation power density, respectively. The thresholds for lasing estimated from Fig. 4d are around 4 kW/cm^2^, 10 kW/cm^2^, 22 kW/cm^2^ for the peaks at 522 nm, 492 nm and 443 nm, respectively. There exists a slight difference in threshold for lasing compared with those obtained earlier under the conditions of using a CW diode laser as an optical pumping source. This can be attributed to the reduction of thermal effects due to the utilisation of a pulsed laser for the TRPL measurements.

The appearance of the ultra-fast decay component in conjunction with the super-linear increase in emission intensity and the sudden reduction in the FWHM of the emission peak provide solid evidences for the lasing behaviour^17–19, 24–29^.

**Figure 5:**
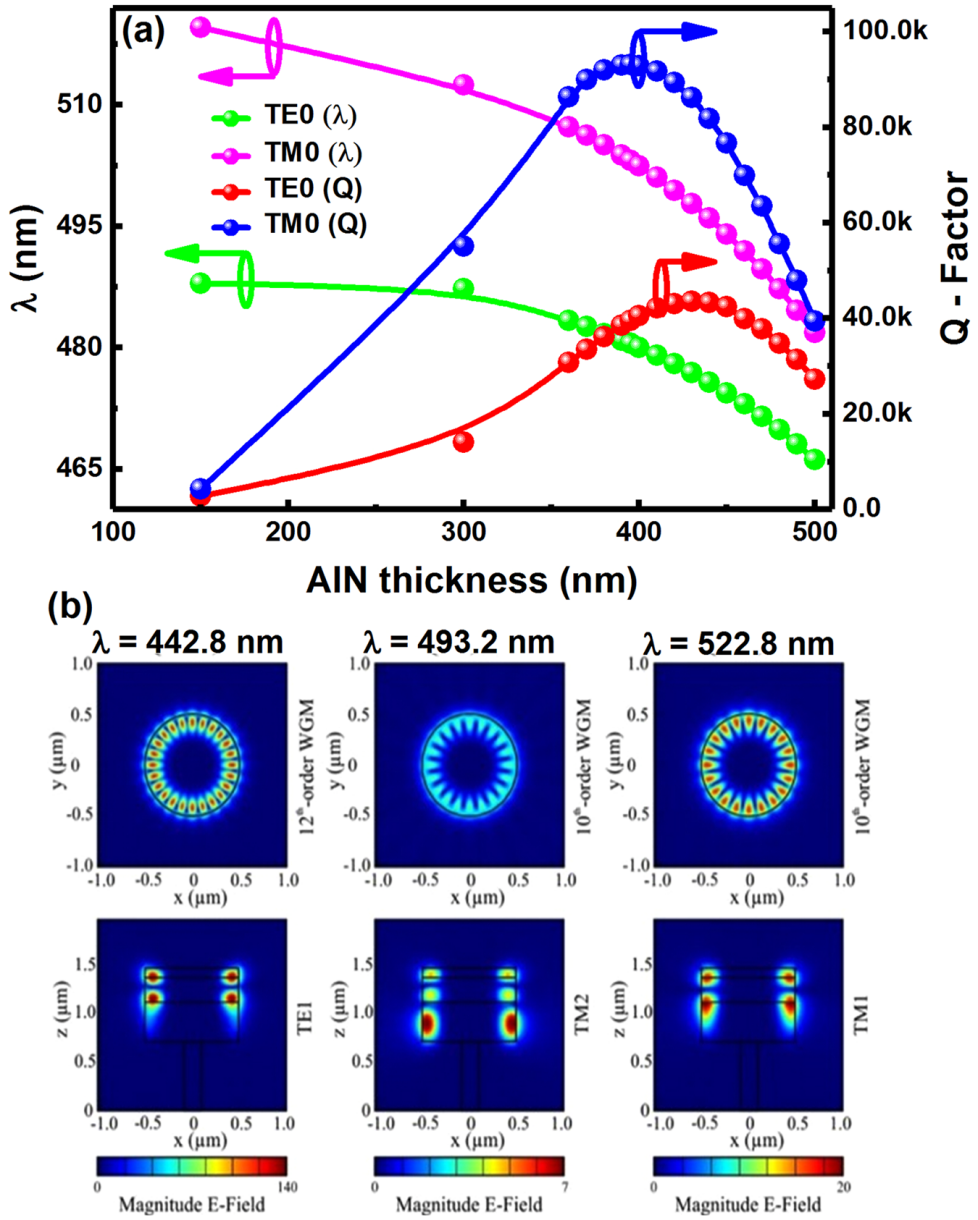
(**a**) Simulated Q-factors for the fundamental modes of both TE and TM as a function of AlN buffer thickness and simulated wavelengths of both the fundamental TE and TM modes as a function of AlN layer thickness; (**b**) Electrical field distribution for the three lasing peaks along the horizontal (i.e., X-Y plot) and vertical (X-Z) directions labelled as (**a**,**b**, and **c**), respectively.

As mentioned previously, it is crucial to make a proper design on AlN buffer thickness in order to effectively suppress vertical modes. In the meantime, a proper design in AlN buffer thickness is also necessary in order to achieve highest Q factors for each lasing peak. From the point of view of epitaxy, it is ideal to have a thick AlN buffer layer in order to improve the crystal quality of a sample grown on a large lattice-mismatched silicon substrate. However, in order to effectively suppress vertical modes, the thickness of the AlN buffer needs to be minimised. A proper AlN buffer layer is required in order to meet both requirements. Figure 5a shows the simulated Q factors for both the fundamental transverse-electric *(TE)* and the fundamental transverse-magnetic *(TM)* modes plotted as a function of AlN layer thickness. It is clear that an AlN buffer layer with a thickness of approximately 400 nm leads to the highest Q-factors for both the TE and TM fundamental modes. Furthermore, the wavelengths of both the fundamental TM and TE modes decrease with increasing AlN buffer thickness. Figure 5b shows the distribution of the electrical fields for the three lasing modes, demonstrating the WG resonances from the top-view mode profiles and effective suppression of the vertical modes within the GaN region as a result of the utilisation of the AlN buffer with a proper thickness.

In summary, we have reported a room-temperature optically pumped multi-colour laser based on an InGaN/GaN microdisk laser with an undercut structure, which was fabricated by means of a combination of a cost-effective microsphere approach with subsequent dry etching and chemical etching processes. The optically pumped lasing was achieved at 443 nm, 492 nm and 522 nm in cw mode simultaneously, forming multi-color lasing, where their corresponding thresholds for lasing are around 27 kW/cm^2^, 15 kW/cm^2^ and 5 kW/cm^2^, respectively. This is the first report of a multi-colour laser with a low threshold using III-nitride semiconductors so far, potentailly paving the way for achieving electrically injected lasing. The lasing behaviour of our micro-disk laser has been confirmed by optical pumping power dependent measurements and time resolved micro-PL measurements. A 3D FDTD simulation has been used to design and engineer the AlN buffer layer in order to effectively suppress the vertical modes and then enhance the confinement within the cavity region.

## Methods

### Fabrication of microdisk lasers

The microdisks were fabricated from an InGaN/GaN MQW epi-wafer, which was grown by MOCVD on an (111) silicon substrate. The epi-wafer consists of a 400 nm AlN buffer layer and then a 250 nm GaN layer, followed by 3 pairs of In_0.18_Ga_0.82_N/GaN MQWs and then 5 pairs of In_0.28_Ga_0.72_N/GaN MQWs, where the thicknesses of the quantum well and the barrier are 2.5 and 10 nm respectively. The epi-wafer was then fabricated into single microdisk structures by a combination of a silica microsphere lithography approach and subsequent dry/etching processes. Initially, the silica micro-particles with a diameter of approximately 1 μm diluted in DI water with 1:50 concentration were deposited directly on the surface of the epi-wafer using a spin coating method at a high rotation speed (7000 rpm), serving as micro masks for subsequent dry-etching. A standard inductive couple plasma (ICP) technique was used to etch the InGaN/GaN epilayer in order to form sparsely distributed micropillars. The silica microspheres were then removed from the top of each micro-pillar simply by using an ultrasonic bath. Finally, a KOH wet etching method was then employed to undercut the micro-disk and then introduce an air gap, which will isolate the micro-disk region from the silicon substrate. A large air-gap (>1 μm) with a tiny post remaining to mechanically support the micro-disk was formed under the micro-disk region, significantly enhancing the optical confinement along the vertical direction and thus minimizing any optical losses to the silicon substrate. A further surface treatment involving the utilization of hot nitric acid has been developed in order to remove any residual etchants and cure any damage generated during the ICP dry etching process.

Lasing spectral measurements have been performed using a commercial WITec confocal microscope. A 375 nm continuous wave (CW) diode laser is used as an optical pumping source and the system is equipped with a 300 mm Princeton instruments monochromator (SP2300i) and an air-cooled charge coupled device (CCD). An objective lens (100×, NA = 0.95) is used to focus the laser beam down to a spot with a diameter of ~220 nm. The emission is dispersed by the monochromator with a resolution of ~0.1 nm. An optical fiber with a diameter of 10 µm acts as a pinhole, thus allowing the emission to be collected only where the excitation is performed. The system is equipped with a high-resolution x-y-z piezo-stage to individually address and examine microdisk lasers. All the measurements have been performed at room temperature.

Time-resolved micro-photoluminescence measurements have been carried out in a micro-PL system, where a 375 nm pulsed diode laser is used as an optical pumping source and the system is equipped with a monochromator (Horiba iHR550) and an air-cooled CCD. An objective lens (50×, NA = 0.43) was used to focus the laser beam down to a spot with a diameter of ~2 μm. The luminescence is dispersed by the monochromator with a resolution of 0.01 nm. For the μ-TRPL measurements, a time-correlated single photon counting (TCSPC) system is used, and the emission is detected by a Hamamatsu hybrid photon counting PMT. The response time of the system is 150 ps.

A three-dimensional finite difference time domain (FDTD) approach has been used to simulate the microdisk structure using commercial-grade software [Lumerical Solutions, Inc. http://www.lumerical.com/tcad-products/fdtd/]. A dipole source with emission wavelengths from 370 to 720 nm was vertically centered in the plane of the microdisk quantum wells and 10 nm inside the edge of the microdisk. All the geometrical data used for the simulation are from the SEM measurement of our microdisk. The diameter and height of the microdisk used for the simulation are 1030 nm and 750 nm, respectively. Other parameters for the simulation include a 400 nm thick AlN buffer layer (*n* = 2.1), a 250 nm thick bulk GaN layer (*n* = 2.44) and a 100 nm thick refractive-index weighted average layer representing the QW region (*n* = 2.47). For the silicon post below the microdisk, a diameter and a height of 260 nm and 700 nm have been used, respectively. There is a minimum of ten mesh cells per wavelength in the simulation that is run for 5000fs. Frequency-domain power monitors have been used to record the emission profile over the simulation region, which is surrounded by perfectly matched layer absorbing boundaries on all sides. The simulated spectra are collected by a grid of 12 time-monitors placed both inside and outside the microdisk structure, accurately calculating the envelope of the time-domain field signal. Each resonant peak is isolated in the frequency domain using a Gaussian filter, and by taking the inverse Fourier transforms the time decay have been calculated separately for each peak. The slope of the time decay is used to calculate the Q-factor of each resonance.

## Electronic supplementary material

Supplementary info
