# Supplementary material for: Monolithically multi-color lasing from an InGaN microdisk on a Si substrate
Source: Sci Rep. 2017 Aug 30;7:10086. doi: 10.1038/s41598-017-10712-4 (PMC5577231; doi:10.1038/s41598-017-10712-4)
Supplement: Supplementary file 1 — Supplementary info [file 41598_2017_10712_MOESM1_ESM.pdf]

Monolithically multi-color lasing from an InGaN microdisk on a Si substrate

M. Athanasiou,[a] R. M. Smith,[a] J. Pugh,[b] Y. Gong,[a] M. J. Cryan[b] and T. Wang [a,*]

[a] Department of Electronic and Electrical Engineering, University of Sheffield, United Kingdom

[b] Department of Electrical and Electronic Engineering, University of Bristol, United Kingdom

*E-mail: t.wang@sheffield.ac.uk

Supplementary information

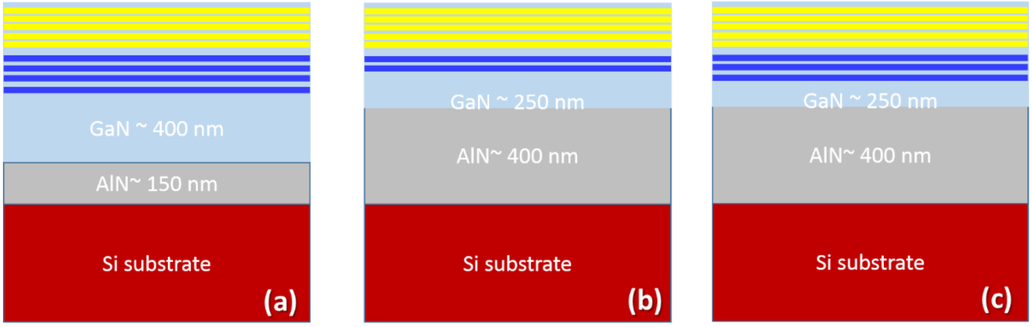

In order to optimise and balance the emission from the blue and green/yellow multiple quantum wells (MQWs) a series of samples have been grown as shown in **Fig. S1(a) to (c).** The number of pairs of both blue and green/yellow MQWs have been changed. Additionally, the thickness of the AlN buffer layer has been optimised in order to improve the vertical confinement.

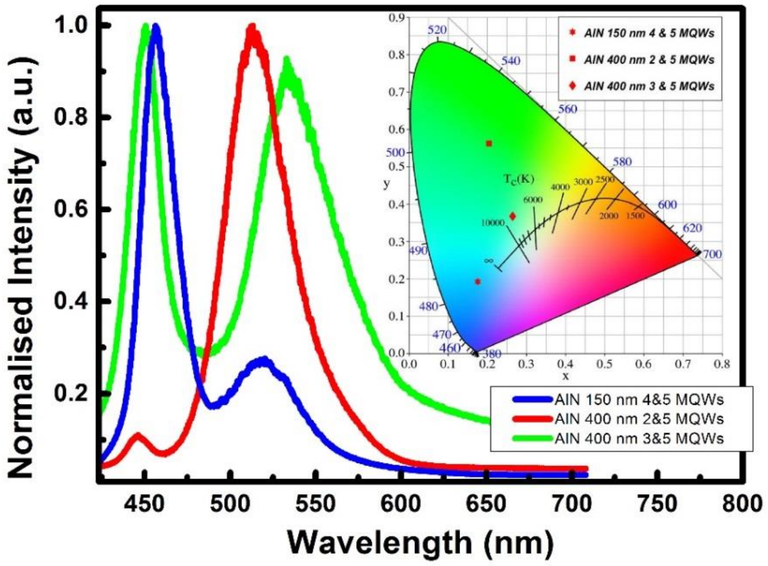

Fig. S2 shows the normalised PL spectra of the as-grown samples as described in **Fig.S1**. Inset shows an indication of CIE plot for the samples measured in a micro-PL set up.

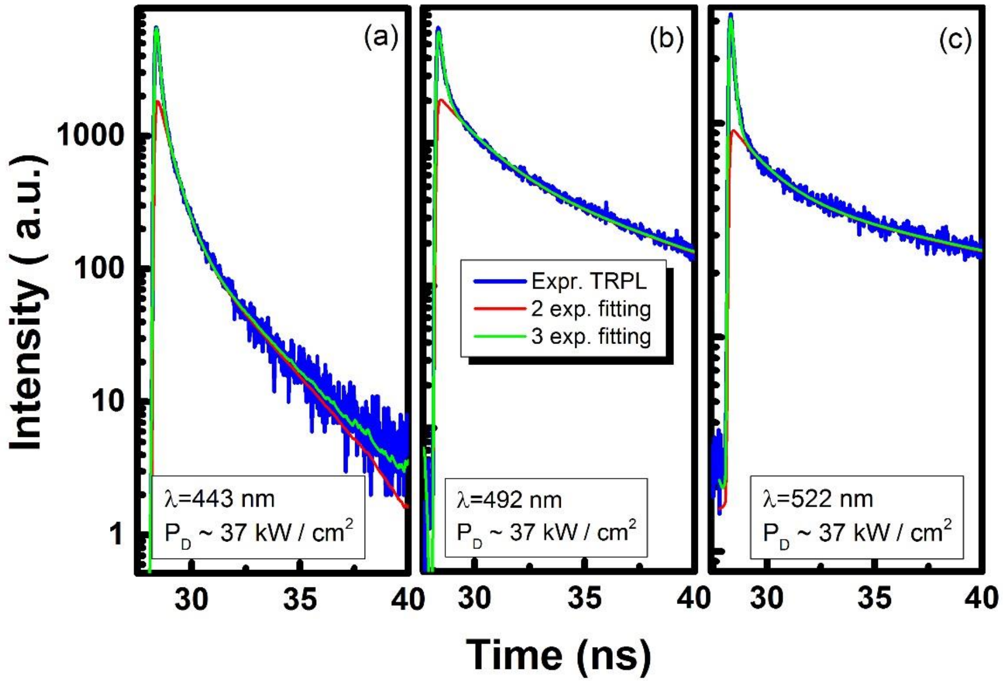

Fig. S3 shows the TRPL decay traces measured on the three lasing peaks under lasing conditions at an excitation power density of 37 kW/cm^2. The red and green lines are the fitted decay traces using the bi-exponential and tri-exponential decays model, respectively.

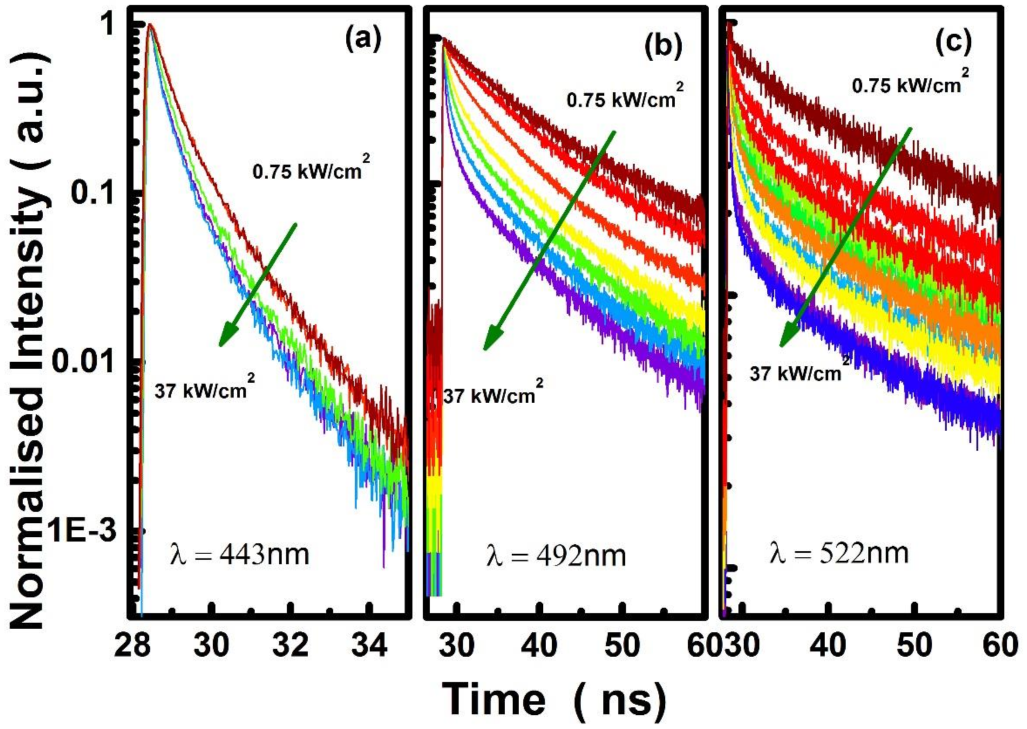

Fig. S4 shows the normalised TRPL decay traces as function of excitation power density for the three lasing peaks at 443 nm, 492nm and 522 nm, demonstrating a sudden drop in PL decay time when the excitation power exceeds the lasing threshold in each case.

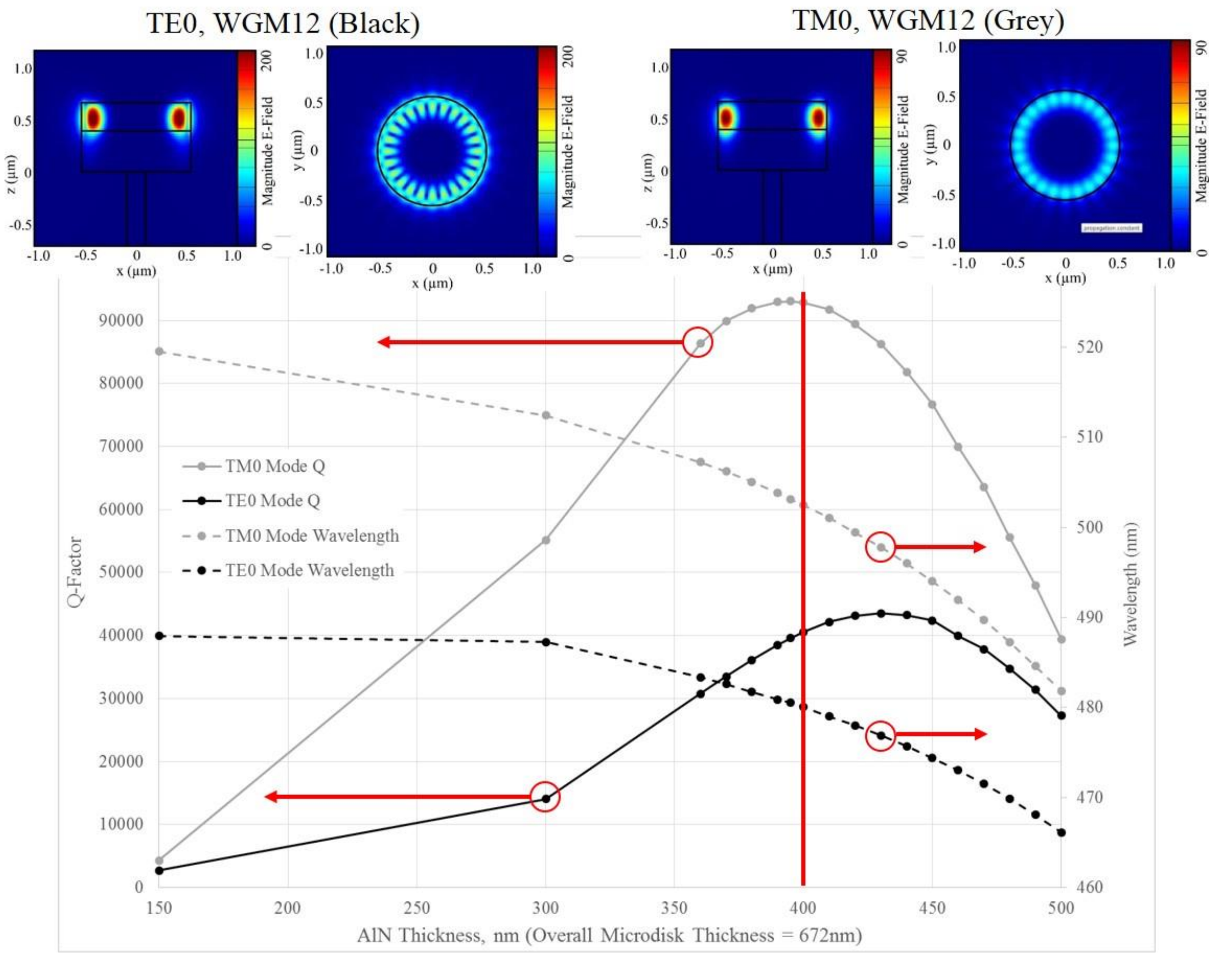

Fig. S5 shows the lumerical FDTD modelling of a micro disk with a microdisk radius of 530 nm and a pillar radius of 50 nm. The AlN layer thickness has been optimised in order to enhance the optical confinement in the GaN region. An optimal AlN layer with a thickness of 400 nm has been determined by modelling the Q-factor of modes and the optical confinement within the structure.

In order to reduce the simulation time required, two-fold symmetry is used in the plane of the quantum wells. The Q-factor should be determined by the slope of the envelope of the decaying signal. Each resonance peak is isolated in the frequency domain using a Gaussian filter, then taking the inverse Fourier Transform to calculate the time decay separately for each peak. The slope of the time decay is then used to calculate the Q-factor from equation,

$$Q = \frac{-2\pi f_R \log_{10}(e)}{2m}$$

Where f_R is the resonant frequency of the mode, and m is the slope of the decay in SI units.

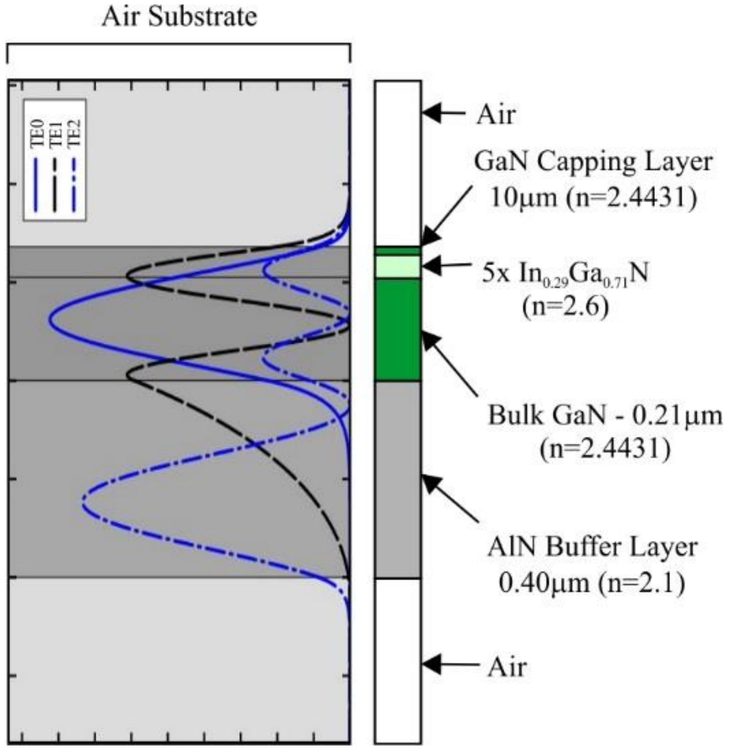

Fig. S6 shows the results of the OMS mode solver (http://www.computational-photonics.eu/oms.html), demonstrating the suppression of higher order modes and the confinement of the fundamental and the 1st higher order mode in the GaN layer region if a 400nm AlN buffer is used

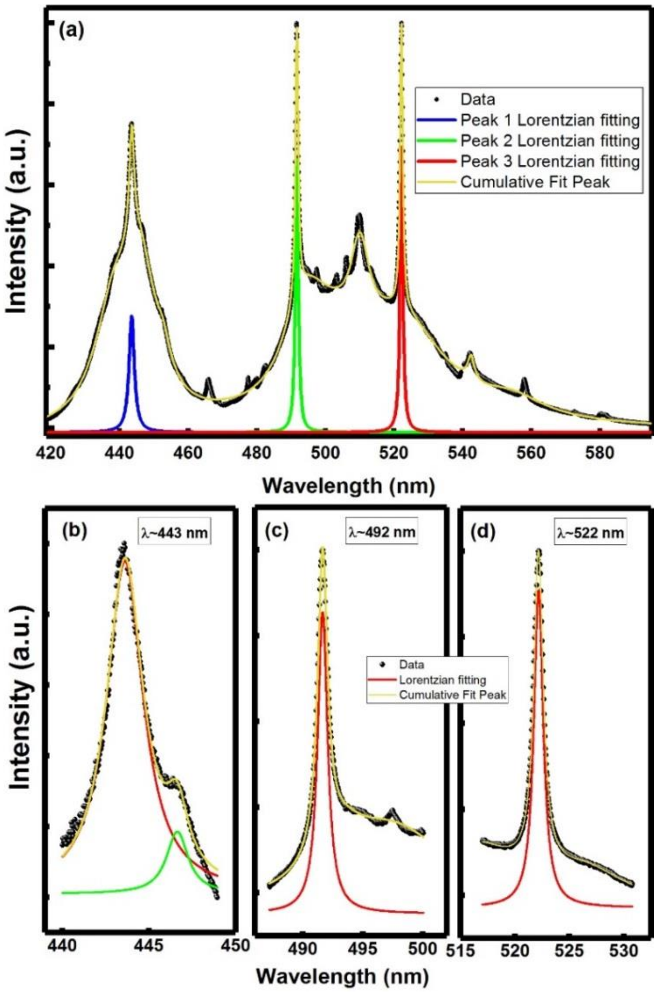

Fig. S7 shows Lorentzian fitting on the three lasing peaks at 443, 492, and 522nm, respectively.

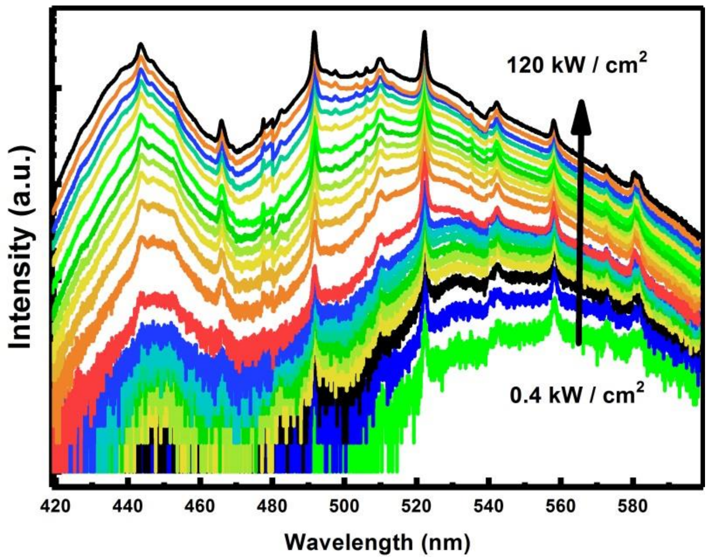

Fig. S8 shows the lasing spectra as a function of excitation power density (originally from Figure 2) plotted on a log scale in order to observe the WGMs clearly below the threshold.

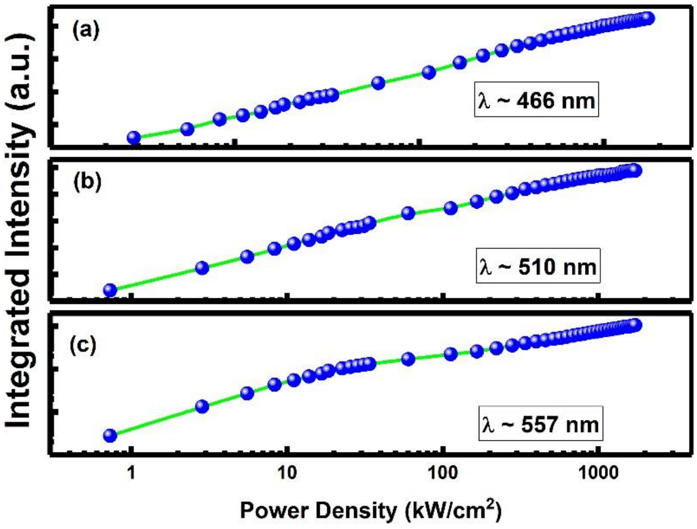

Fig. S9 (a) to (c) shows L-I characteristics of the non-lasing peaks at 466 nm, 510 nm and 557 nm, respectively.

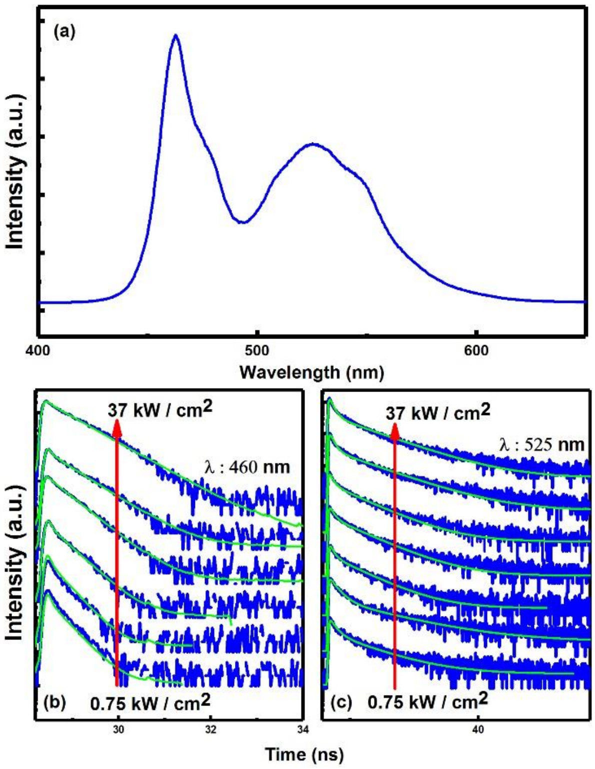

Fig. S10 (a) shows the μ-PL spectrum of the as-grown sample measured at room temperature, exhibiting two emission peaks at 460 and 525 nm, respectively; (b) TRPL decay traces as a function of excitation power density measured on these two peaks. A bi-exponential model (labelled as green colour) can be used to fit them very well.

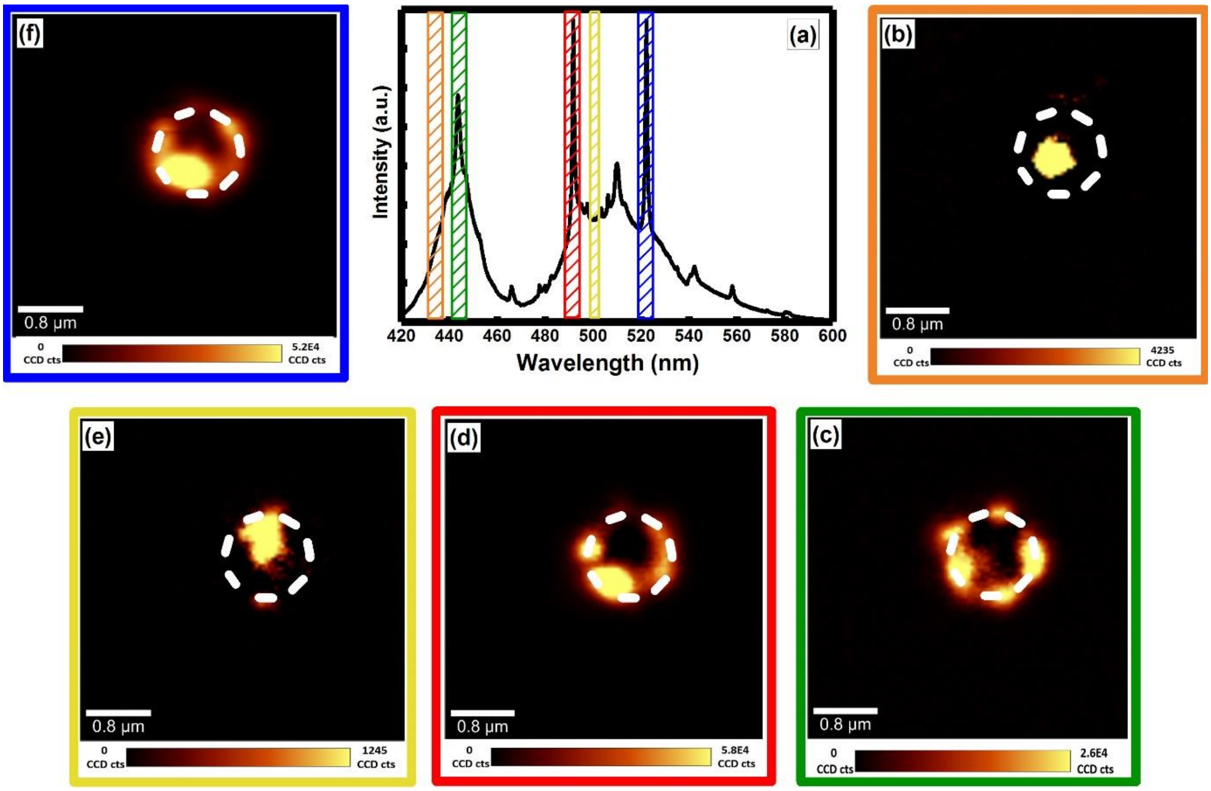

Fig. S11 (a) shows the con-focal PL mapping under lasing conditions, indicating the integrated areas for the confocal PL mapping centred at (b) 433 nm (c) 443 nm (d) 492 nm (e) 500 nm and (f) 522 nm, respectively. The on-resonances confocal PL mapping shows the light coupling into WGM modes, which circulates around the periphery of the micro-disk, where in the off-resonance cases the emission is mainly from the centre of the micro-disk.
